# Distinguishing Polymorphonuclear Leukocyte-Predominant Tuberculous Pleural Effusion from Parapneumonic Effusion in Patients with Elevated Serum C-Reactive Protein

**DOI:** 10.3390/jcm15186976

**Published:** 2026-09-09

**Authors:** Seongwon Shin, Chi Young Jung, Chang Ho Kim, Jaehee Lee

**Affiliations:** 1 Department of Pulmonology, Kyungpook National University Hospital, Daegu 41944, Republic of Korea; kei010501@gmail.com; 2 Department of Pulmonology, Daegu Catholic University Medical Center, Daegu 42472, Republic of Korea; jcy2475@cu.ac.kr; 3 Department of Internal Medicine, School of Medicine, Kyungpook National University, Daegu 41944, Republic of Korea

**Keywords:** tuberculous pleural effusion, parapneumonic effusion, adenosine deaminase, C-reactive protein, polymorphonuclear leukocyte, computed tomography

## Abstract

**Background:** Tuberculous pleural effusion (TPE) occasionally presents with polymorphonuclear leukocyte (PMN)-predominant pleural fluid (PF) and elevated serum C-reactive protein (CRP), mimicking parapneumonic effusion (PPE). We investigated factors distinguishing TPE from PPE in this diagnostically challenging setting. **Methods:** We retrospectively reviewed consecutive patients with confirmed TPE or PPE at a tertiary referral center (2010–2024). Patients with serum CRP ≥ 10 mg/dL were classified according to PF PMN-predominance. Independent discriminators between PMN-predominant TPE and PPE were identified using multivariable Firth logistic regression. **Results:** Among 414 patients with confirmed TPE, 108 (26%) had serum CRP ≥ 10 mg/dL, including 20 with PMN-predominance and 88 without. Anti-tuberculosis treatment was modestly delayed in the PMN-predominant group (*p* = 0.017). These 20 patients were compared with 335 PPE patients showing the same CRP/PMN profile. Pulmonary nodular lesions on chest computed tomography and PF adenosine deaminase (ADA) were independent discriminators. A composite two-tier rule—nodular lesions present or PF ADA > 80 IU/L if absent—achieved an AUROC of 0.925, 80.0% sensitivity, and 86.2% specificity. **Conclusions:** In patients with PMN-predominant pleural effusion and elevated serum CRP, this simple, readily available rule may help identify patients warranting early pleural biopsy and heightened suspicion for TPE before microbiological confirmation.

## 1. Introduction

Serum C-reactive protein (CRP), a major acute-phase protein that increases in response to infection and tissue injury, is one of the most widely used systemic inflammatory markers in clinical practice [1,2]. Marked CRP elevation is commonly observed in severe bacterial infection, whereas non-bacterial inflammatory disorders generally produce lower or more variable CRP concentrations [3,4]. CRP concentrations ≥ 10 mg/dL are generally considered suggestive of severe bacterial infection, although CRP alone lacks sufficient specificity to establish a bacterial etiology [5,6,7].

In patients with pleural effusion, the pleural fluid (PF) leukocyte differential provides important etiologic information during the initial evaluation. When polymorphonuclear leukocyte (PMN) predominance is accompanied by markedly elevated serum CRP, the findings raise strong suspicion for parapneumonic effusion (PPE) and often prompt empirical antibacterial treatment [8,9].

However, this diagnostic approach may be misleading in a small but clinically important subset of patients because tuberculous pleural effusion (TPE) can exhibit PMN predominance in approximately 10–15% of cases, particularly during the early phase of disease [10,11,12]. Importantly, marked serum CRP elevation may also occur in a subset of patients with TPE despite the absence of pyogenic bacterial infection [13,14]. When PF PMN predominance and marked serum CRP elevation co-occur, TPE may be clinically indistinguishable from PPE at initial presentation, potentially resulting in an inaccurate initial diagnosis. Rapid microbiological tests, including PF acid-fast bacilli (AFB) smear and nucleic acid amplification tests for *Mycobacterium tuberculosis* (MTB), are negative in the majority of confirmed TPE cases, whereas mycobacterial culture results may not become available for several weeks [12,15,16]. In the absence of microbiological confirmation, definitive diagnosis may require pleural biopsy to identify granulomatous inflammation or microbiological evidence of MTB in pleural tissue [8,15,17]. However, the clinical indications for early pleural biopsy in this setting remain poorly defined.

An additional diagnostic challenge is the limited specificity of PF adenosine deaminase (ADA) in PMN-predominant effusions, as complicated PPE and empyema may also produce elevated ADA levels [18,19]. These overlapping diagnostic features further complicate differentiation in this subgroup, despite its substantial therapeutic implications: TPE requires anti-tuberculosis (TB) treatment, whereas PPE requires antibacterial therapy, with pleural drainage when indicated. Delayed diagnosis may postpone anti-TB treatment and prolong potential transmission in patients with concomitant pulmonary involvement [11].

Prior studies have examined PF biomarkers and radiological findings to differentiate TPE from PPE, but these have largely focused on TPE in general or on lymphocyte-predominant cohorts, and have not specifically addressed the subgroup with PMN-predominant TPE accompanied by markedly elevated serum CRP [20,21]. Consequently, no established criteria currently guide decisions regarding early pleural biopsy in this clinical scenario. To address this gap, the present study aimed to identify independent predictors that distinguish PMN-predominant TPE with elevated serum CRP from PPE and to develop a practical risk-stratification approach based on radiological findings and biochemical profiles to guide decisions regarding early pleural biopsy. In addition, to explore whether PMN-predominant TPE itself is associated with delayed treatment initiation, we performed a secondary comparison with a cohort of non-PMN-predominant TPE.

## 2. Materials and Methods

### 2.1. Study Design and Setting

This was a retrospective cohort study conducted at a tertiary referral hospital in Daegu, South Korea: Kyungpook National University Hospital (KNUH). All consecutive patients diagnosed with confirmed TPE or PPE between January 2010 and December 2024 were reviewed. The study was approved by the Institutional Review Boards of KNUH, and the requirement for written informed consent was waived owing to the retrospective nature of the study.

### 2.2. Patient Cohorts and Group Definitions

Patients with TPE were identified from the hospital TB notification registry. TPE was considered confirmed if one or more of the following criteria were met: (1) positive culture for MTB from PF, pleural tissue, sputum, or bronchial aspirate; (2) histological evidence of chronic granulomatous inflammation on pleural biopsy with clinical response to anti-TB treatment; or (3) positive MTB-polymerase chain reaction (PCR) in PF or pleural tissue together with compatible clinical findings. Patients without microbiological or histological confirmation were excluded.

PPE was diagnosed in patients with exudative pleural effusion associated with bacterial pneumonia, lung abscess, or bronchiectasis, without microbiological evidence of MTB in serial PF, sputum, or bronchial aspirate specimens. All patients classified as PPE received antibacterial therapy, and available follow-up records demonstrated clinical and radiological resolution without subsequent evidence of TB during at least 3 months of follow-up. Patients with grossly purulent pleural fluid (empyema) at initial thoracentesis were excluded from both cohorts.

Confirmed TPE patients were further classified according to serum CRP concentration (measured in mg/dL; ≥10 mg/dL corresponds to ≥100 mg/L) and PF differential cell count obtained at initial thoracentesis as follows: Group A, CRP ≥ 10 mg/dL and PMN > 50%; Group B, CRP ≥ 10 mg/dL and PMN ≤ 50%. Group C comprised PPE patients with CRP ≥ 10 mg/dL and PMN > 50%. The CRP threshold of ≥10 mg/dL, commonly associated with bacterial infection in the previous literature, was applied uniformly across all study groups [3,6,7].

### 2.3. Data Collection

Clinical, laboratory (including serum, PF, and microbiological findings), and radiologic data were extracted from electronic medical records. Clinical variables included age, sex, previous TB history, fever, weight loss (>10% during the preceding 6 months), and chest pain. Blood tests, including white blood cell (WBC) count, serum CRP, and serum lactate dehydrogenase (LDH), were obtained within 24 h of the initial thoracentesis. PF analyses included pH, ADA, PMN%, glucose, total protein, and LDH. Microbiological data included AFB smear, MTB-PCR, mycobacterial culture, and bacterial culture results from PF, sputum, and bronchial aspirate specimens. Computed tomography (CT) of chest was performed before or within 48 h after the initial thoracentesis. Images were independently reviewed by a board-certified radiologist and a pulmonologist who were blinded to the final diagnosis; discordant interpretations were resolved by consensus with a third physician. CT variables included PF loculation and lung parenchymal lesions (cavitary lesions, pulmonary nodular lesions, and consolidation). Pulmonary nodular lesions were defined as discrete nodules (≤20 mm) or micronodules including centrilobular micronodules and tree-in-bud opacities [22]. Only findings from the initial thoracentesis were included in the analyses.

PF pH was measured using a Radiometer ABL blood-gas analyzer (Radiometer, Copenhagen, Denmark); the specific analyzer model was updated during the study period as part of routine laboratory equipment turnover. PF ADA was measured using an automated colorimetric assay kit (Runpia Liquid ADA; Kyokuto Pharmaceutical Industrial Co., Ltd., Tokyo, Japan).

### 2.4. Statistical Analysis

Continuous variables are reported as median [interquartile range (IQR)] and were compared between groups using the Mann–Whitney U test. Categorical variables are expressed as n/N (%) and were compared using Fisher’s exact test or the chi-square test as appropriate. Pairwise comparisons across the three groups were not adjusted for multiple comparisons.

To identify independent discriminators between Group A and Group C, univariable and multivariable logistic regression analyses were performed using Firth’s penalized maximum likelihood method, implemented in-house in Python (NumPy/SciPy) following Firth [23] and Heinze and Schemper [24]. Variables were selected based on clinical relevance and univariable association with the outcome and were assessed for multicollinearity before inclusion in the multivariable model. Analyses were performed on a complete-case basis.

Candidate models were compared using the area under the receiver operating characteristic curve (AUROC), with formal comparison between correlated ROC curves performed using the DeLong test; internal validation was assessed via Harrell’s bootstrap optimism correction. Optimal cutoffs for continuous predictors were determined using the Youden index (sensitivity + specificity − 1). The composite rule was derived by dichotomizing the model-predicted probability at its Youden-optimal cutoff and algebraically simplifying the resulting decision boundary into a two-tier rule based on pulmonary nodular lesions and, secondarily, PF ADA.

Diagnostic performance of independent predictors was assessed individually and as part of the composite model, using sensitivity, specificity, positive predictive value (PPV), negative predictive value (NPV) and AUROC. All tests were two-tailed, with *p* < 0.05 considered significant. Statistical analyses were performed using Python version 3.12 (Python Software Foundation, Wilmington, DE, USA). Generative artificial intelligence (AI)-assisted technology (Claude, Anthropic) was used to provide guidance on selected aspects of the statistical methodology and analytical procedures. All statistical analyses were performed and verified by the authors, who retained full responsibility for the selection of statistical methods, interpretation of the results, and scientific conclusions.

## 3. Results

### 3.1. Study Population

**Figure 1:**
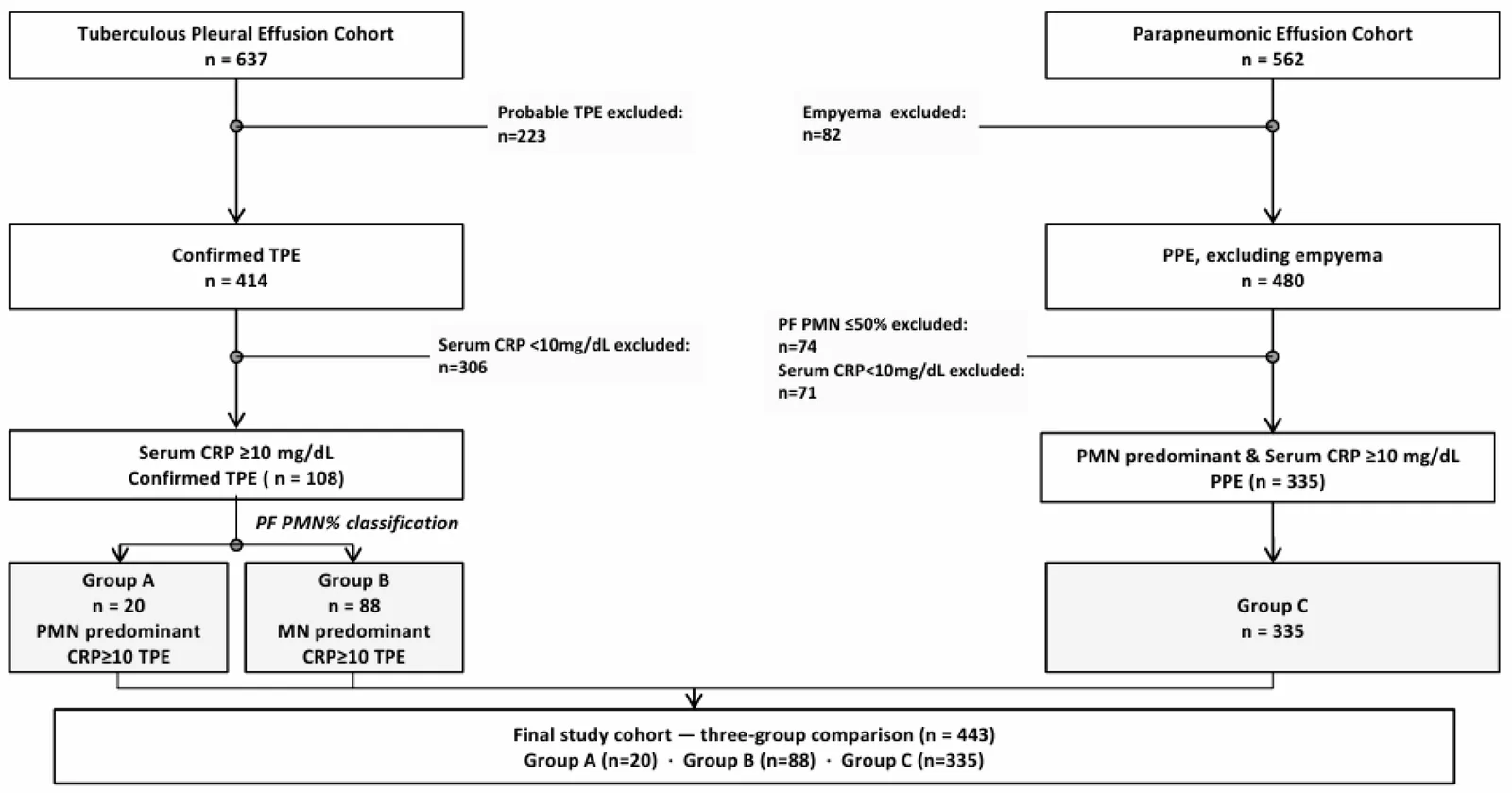
Flow diagram of patient selection for Group A (PMN-predominant tuberculous pleural effusion [TPE] with serum C-reactive protein [CRP] ≥ 10 mg/dL), Group B (non-PMN-predominant TPE with serum CRP ≥ 10 mg/dL), and Group C (PMN-predominant parapneumonic effusion [PPE] with serum CRP ≥ 10 mg/dL).

During the study period, 637 consecutive patients were diagnosed with TPE, of whom 414 met the predefined criteria for confirmed TPE. Among these 414 patients, 108 (26%) had serum CRP ≥ 10 mg/dL and were further classified according to the PF differential cell count into Group A (PMN > 50%, *n* = 20) and Group B (PMN ≤ 50%, *n* = 88). For the PPE cohort, 562 consecutive patients were reviewed; after excluding cases with empyema, 335 patients with both PMN > 50% and serum CRP ≥ 10 mg/dL constituted Group C (Figure 1).

Of the 20 patients in Group A, 17 (85%) were confirmed by positive MTB culture from at least one specimen source, two (10%) by histological chronic granulomatous inflammation on pleural biopsy with clinical response to anti-TB treatment, and one (5%) by positive MTB-PCR on PF in conjunction with clinical response to anti-TB treatment. Microbiological confirmation based on PF alone was limited, with positive PF culture, MTB-PCR, and AFB smear in only 35%, 15%, and 5% of Group A patients, respectively.

### 3.2. Comparison Between Group A and Group B

To characterize the clinical presentation and timing of treatment in patients with PMN-predominant TPE, Groups A and B were compared. Demographic characteristics, including age, sex, and previous TB history, did not differ significantly between Groups A and B (Table 1).

Among clinical symptoms, weight loss was more frequent in Group B than in Group A (26% vs. 5%; *p* = 0.041). Fever and chest pain did not differ significantly between the two groups. Blood test results, including WBC, CRP, and LDH, did not differ significantly between the two groups. As expected, PF PMN% differed significantly because of the predefined group definitions (Group A 60.0% [52.2–85.8] vs. Group B 10.0% [2.8–27.4]; *p* < 0.001). PF ADA tended to be higher in Group B (90.0 [70.3–111.1] vs. 72.2 [53.4–92.4] IU/L; *p* = 0.053). PF protein was marginally higher in Group B (4.7 [4.3–5.2] vs. 4.3 [3.6–5.0] g/dL; *p* = 0.075). All other PF parameters were similar between groups. None of the radiological findings, including loculated effusion, cavity, pulmonary nodular lesion, or consolidation, showed significant differences. The interval between thoracentesis and initiation of anti-TB treatment was significantly longer in Group A than in Group B (3.0 [1.0–7.2] vs. 1.0 [0.0–3.0] days; *p* = 0.017).

### 3.3. Comparison Between Group A and Group C

Demographic variables were similar across Groups A and C (Table 1). Among clinical symptoms, chest pain tended to be less frequent in Group A (55% vs. 77%; *p* = 0.056), whereas fever and weight loss were not significantly different between the groups.

Blood WBC count was significantly lower in Group A than in Group C (9310 [6258–10,872] vs. 14,470 [11,370–19,175] cells/μL; *p* < 0.001), as was serum CRP (14.0 [11.4–17.0] vs. 22.0 [16.2–28.7] mg/dL; *p* < 0.001).

Regarding PF analysis, Group A showed a higher PF pH (7.37 [7.34–7.42] vs. 7.20 [7.00–7.34]; *p* < 0.001) and higher PF ADA (72.2 [53.4–92.4] vs. 39.1 [29.6–52.2] IU/L; *p* < 0.001) than Group C. Despite both groups having PMN > 50% by definition, the degree of PMN% was significantly lower in Group A (60.0% [52.2–85.8] vs. 88.9% [77.0–95.0]; *p* < 0.001). PF protein, glucose, and LDH were not significantly different between the groups.

Loculated effusion was less frequent in Group A than in Group C (25% vs. 74%; *p* < 0.001), whereas pulmonary nodular lesions were more frequent in Group A (70% vs. 7%; *p* < 0.001). Cavity and consolidation did not differ significantly between the two groups.

### 3.4. Logistic Regression Analysis: Discriminators Between Group A and Group C

Pulmonary nodular lesions showed the strongest univariable association with Group A (odds ratio [OR], 29.570; 95% CI, 10.637–82.197; *p* < 0.001) (Table 2). Loculated effusion was significantly negatively associated with Group A (OR, 0.127; 95% CI, 0.046–0.347; *p* < 0.001). Other significant univariable predictors included blood WBC count (OR, 0.835; 95% CI, 0.752–0.927; *p* < 0.001), serum CRP (OR per mg/dL, 0.824; 95% CI, 0.749–0.908; *p* < 0.001), PF pH (OR, 150.8; 95% CI, 7.7–2941.9; *p* < 0.001), PF PMN% (OR, 0.931; 95% CI 0.903–0.960; *p* < 0.001), and PF ADA (OR, 1.017; 95% CI, 1.007–1.027; *p* = 0.001).

In the multivariable Firth logistic regression model, only PF ADA (adjusted OR 1.053, 95% CI 1.029–1.079; *p* < 0.001) and pulmonary nodular lesions (adjusted OR 21.853, 95% CI 5.210–91.652; *p* < 0.001) remained independent discriminators between Groups A and C. None of the other variables retained statistical significance after adjustment.

The diagnostic performance of the two independent predictors is summarized in Table 3. PF ADA > 55 IU/L yielded a sensitivity of 70.0% and specificity of 78.5% (AUROC, 0.798), while pulmonary nodular lesions showed a similar sensitivity (70.0%) but higher specificity (93.0%; AUROC, 0.817). Combining these two predictors via multivariable Firth logistic regression yielded a composite score with improved discrimination (optimism-corrected AUROC, 0.925; 95% CI, 0.881–0.974); algebraic simplification of this model reduced its decision boundary to a simple two-tier rule, classifying patients as high-risk for TPE if pulmonary nodular lesions were present on CT or if PF ADA exceeded 80 IU/L when nodular lesions were absent. This composite rule achieved a sensitivity of 80.0% (95% CI, 58.4–91.9%) and specificity of 86.2% (95% CI, 82.1–89.5%), significantly outperforming PF ADA alone (DeLong test, *p* = 0.016) and pulmonary nodular lesions alone (*p* = 0.003). Within patients with nodular lesions on CT, further raising the PF ADA threshold to >115 IU/L increased specificity to 100% (334/334), although this markedly reduced sensitivity to 10.0% (2/20).

In a sensitivity analysis re-including 48 patients with empyema meeting the same PMN-predominance and CRP ≥ 10 mg/dL criteria (excluded from the primary analysis), median PF ADA in this subgroup was 203.7 IU/L (IQR, 123.2–334.0). When these patients were added to Group C, the specificity of the composite rule decreased from 86.2% to 76.4% (sensitivity unchanged at 80.0%).

## 4. Discussion

The present study addressed the clinically most challenging diagnostic scenario in pleural disease: patients presenting with both serum CRP ≥ 10 mg/dL and PMN-predominant pleural effusion, a combination that strongly favors a diagnosis of PPE in routine clinical practice. To our knowledge, this is the first study specifically designed to evaluate confirmed TPE within this high-CRP, PMN-predominant subset. This study specifically focused on patients with serum CRP ≥ 10 mg/dL because this threshold commonly prompts empirical antibacterial treatment in routine clinical practice [5,6,7]. In multivariable Firth logistic regression analysis, pulmonary nodular lesions on chest CT and PF ADA were identified as independent discriminators. Furthermore, a composite two-tier rule—classifying patients as high-risk for TPE if nodular lesions were present on chest CT, or if PF ADA exceeded 80 IU/L when nodular lesions were absent—showed good overall discrimination (optimism-corrected AUROC, 0.925; sensitivity, 80.0%; specificity, 86.2%), suggesting that these readily available parameters may help identify patients otherwise presumed to have PPE who instead warrant early consideration of TPE.

Among all evaluated variables, pulmonary nodular lesions on CT showed the strongest radiologic discriminatory ability between TPE and PPE, being present in 70% of Group A compared with only 7% of Group C. Because nodular lesions—including centrilobular micronodules and tree-in-bud opacities—represent a typical radiologic manifestation of pulmonary TB [25], their frequent detection in our cohort is an expected consequence of concomitant pulmonary involvement rather than a feature specific to PMN-predominant TPE itself. Consistent with this interpretation, the genuinely challenging diagnostic scenario is isolated TPE without parenchymal findings, in which this predictor is absent by definition. Within the nodule-negative subgroup of our cohort (*n* = 317: 6 TPE, 311 PPE), PF ADA > 80 IU/L identified only 2 of 6 TPE cases (sensitivity 33.3%; specificity 92.6%). Given the very small number of nodule-negative TPE cases, this observation should be regarded as hypothesis-generating rather than a validated estimate of diagnostic performance in this subgroup. In contrast, consolidation did not differ significantly between the two groups (45% vs. 63%; *p* = 0.152; Table 1), suggesting that this finding has limited value in distinguishing TPE from PPE despite similarly high systemic inflammatory responses. This observation also agrees with our previous study, which identified pulmonary nodular lesions—but not consolidation—as an independent discriminator between TPE and PPE in patients with non-lymphocytic pleural effusion and elevated PF ADA [21]. In that earlier study, loculated effusion was also an independent negative predictor of TPE; in the present cohort, although loculated effusion was significantly less frequent in Group A than in Group C on univariable analysis (25% vs. 74%; *p* < 0.001), it did not remain an independent discriminator after multivariable adjustment (adjusted OR, 0.307; 95% CI, 0.062–1.520; *p* = 0.148), suggesting that its discriminatory value may be attenuated once both groups are restricted to a high serum CRP threshold.

Despite previous concerns regarding reduced specificity in PMN-predominant pleural effusions [19], PF ADA remained an independent discriminator after multivariable adjustment in the present study (adjusted OR, 1.053; 95% CI, 1.029–1.079; *p* < 0.001). A cutoff value of >55 IU/L, determined using the Youden index, yielded a sensitivity of 70.0% and specificity of 78.5%. These findings suggest that the diagnostic utility of ADA is reduced but not eliminated in the presence of neutrophilic inflammation. Rather than relying on ADA alone, combining ADA with complementary radiologic findings substantially improved diagnostic accuracy, highlighting the importance of multimodal interpretation in this diagnostically challenging subgroup.

The diagnostic challenge of Group A is further compounded by the limited diagnostic yield of microbiological tests. In our cohort, pleural fluid AFB smear, MTB-PCR, and mycobacterial culture were positive in only 5%, 15%, and 35% of patients, respectively. Reflecting this diagnostic uncertainty, initiation of anti-TB treatment was delayed by a median of 2 days (3 vs. 1 day) in Group A compared with Group B (*p* = 0.017); although statistically significant, this absolute difference is modest. Indeed, this is a broader clinical reality: because culture confirmation requires several weeks and is therefore unavailable during the initial clinical decision-making period, clinicians frequently continue empirical antibiotic therapy while delaying anti-TB treatment [8,9]. Under these circumstances, the composite two-tier rule—classifying patients as high-risk for TPE if nodular lesions are present on CT, or if PF ADA exceeds 80 IU/L when nodular lesions are absent—provides a practical, clinically accessible risk stratification tool that may facilitate earlier consideration of pleural biopsy before definitive microbiological confirmation becomes available [11,15]. Given its high NPV (98.6%) but modest PPV (25.8%) at the observed cohort prevalence, this rule is best applied to identify patients unlikely to have TPE and to support, rather than replace, clinical decision-making regarding early biopsy. Notably, among patients with nodular lesions on CT, further raising the PF ADA threshold to >115 IU/L increased specificity to 100% (334/334), although sensitivity fell to 10% (2/20); given the small sample size, this threshold is best regarded as hypothesis-generating. The coexistence of markedly elevated PF ADA and pulmonary nodular lesions may nonetheless help prioritize early pleural biopsy in situations where microbiological confirmation is unavailable or delayed.

This study has several limitations. First, it was a retrospective, single-center study conducted in an intermediate-TB-burden region, and restricting the TPE group to confirmed cases may additionally have selected for more advanced disease; both factors may limit generalizability, particularly to low-burden settings. Second, the number of patients with PMN-predominant TPE was small, reflecting the rarity of this phenotype; although Firth’s penalized logistic regression and bootstrap resampling were applied to reduce small-sample bias, wider confidence intervals remain inevitable. This instability was most apparent in the exploratory 8-variable model (Table 2), where several coefficients—most notably PF pH—showed extremely wide confidence intervals; these estimates should therefore be interpreted with caution rather than as independently validated risk factors. Additionally, variable selection was not repeated within each bootstrap resample, so the reported optimism-corrected AUROC may not fully capture optimism from the overall model-development process. Third, although the serum CRP ≥ 10 mg/dL threshold used to define all three groups is supported by previous literature as indicative of a high likelihood of bacterial infection, it has not been specifically validated as a cutoff for distinguishing TPE from PPE; the threshold was applied empirically to isolate the clinical scenario most likely to be misdiagnosed as bacterial infection. Fourth, reported specificity applies to differentiation from non-empyematous PPE and is expected to be lower when empyema is included in the differential; similarly, the diagnostically most difficult subgroup—isolated TPE without parenchymal findings—remained underpowered in our cohort (only 6 nodular lesion-negative TPE cases). Finally, the proposed ADA cutoff and composite diagnostic model require external validation in prospective multicenter cohorts before routine clinical implementation.

## 5. Conclusions

In patients presenting with PMN-predominant pleural effusion and serum CRP ≥ 10 mg/dL, TPE can closely mimic PPE and may therefore be associated with delayed diagnosis and treatment. A simple, readily available composite two-tier rule—based on the presence of pulmonary nodular lesions and, secondarily, PF ADA—may serve as a risk-stratification tool in this diagnostically challenging setting, helping identify patients who warrant early pleural biopsy.

## Figures and Tables

**Table 1:** Clinical, laboratory, and radiological characteristics of patients with tuberculous pleural effusion and parapneumonic pleural effusion with serum C-reactive protein ≥ 10 mg/dL, stratified by pleural fluid cellular predominance.

Variable	Group A (*n* = 20) PMN-Predominant TPE	Group B (*n* = 88) Non-PMN-Predominant TPE	Group C (*n* = 335) PMN-Predominant PPE	*p*-Value (A–B)	*p*-Value (A–C)
Demographics					
Age (years)	61.5 [52.8–77.5]	70.0 [47.8–79.2]	64.0 [56.0–75.5]	0.641	0.596
Male sex	16/20 (80%)	63/88 (72%)	273/335 (81%)	0.581	0.774
Previous TB history	3/20 (15%)	8/88 (9%)	37/333 (11%)	0.424	0.484
Symptoms					
Fever	15/20 (75%)	60/88 (68%)	247/335 (74%)	0.788	1.000
Weight loss	1/20 (5%)	23/88 (26%)	15/334 (4%)	0.041	1.000
Chest pain	11/20 (55%)	42/88 (48%)	255/332 (77%)	0.625	0.056
Blood tests					
WBC (cells/µL)	9310 [6258–10,872]	7435 [6102–8990]	14,470 [11,370–19,175]	0.063	<0.001
CRP (mg/dL)	14.0 [11.4–17.0]	13.9 [11.9–16.9]	22.0 [16.2–28.7]	0.953	<0.001
LDH (U/L)	225 [196–309]	242 [209–272]	198 [164–250]	0.935	0.164
Pleural fluid analysis					
pH	7.37 [7.34–7.42]	7.41 [7.34–7.44]	7.20 [7.00–7.34]	0.186	<0.001
WBC (cells/µL)	1360 [344–3264]	1330 [900–2400]	4355 [1221–11,798]	0.695	<0.001
PMN (%)	60.0 [52.2–85.8]	10.0 [2.8–27.4]	88.9 [77.0–95.0]	<0.001	<0.001
Protein (g/dL)	4.3 [3.6–5.0]	4.7 [4.3–5.2]	4.6 [4.0–5.0]	0.075	0.328
Glucose (mg/dL)	95.5 [65.2–149.2]	96.0 [71.0–122.0]	77.0 [27.0–113.5]	0.935	0.142
LDH (U/L)	1030 [402–1604]	782 [434–1403]	1146 [732–1834]	0.436	0.239
ADA (IU/L)	72.2 [53.4–92.4]	90.0 [70.3–111.1]	39.1 [29.6–52.2]	0.053	<0.001
Radiologic findings					
Loculated	5/20 (25%)	28/88 (32%)	247/335 (74%)	0.788	<0.001
Cavity	4/20 (20%)	13/88 (15%)	24/335 (7%)	0.515	0.063
Pulmonary nodular lesion	14/20 (70%)	54/88 (61%)	23/335 (7%)	0.610	<0.001
Consolidation	9/20 (45%)	37/88 (42%)	212/335 (63%)	0.808	0.152
Time from thoracentesis to anti-TB treatment, d	3.0 [1.0–7.2]	1.0 [0–3.0]	–	0.017	–

Data are median [interquartile range] for continuous variables and n/N (%) for categorical variables. TPE = tuberculous pleural effusion; PPE = parapneumonic effusion; PMN = polymorphonuclear leukocyte; TB = tuberculosis; WBC = white blood cell; CRP = C-reactive protein; LDH = lactate dehydrogenase; ADA = adenosine deaminase.

**Table 2:** Logistic regression analysis discriminating polymorphonuclear leukocyte-predominant tuberculous pleural effusion from parapneumonic pleural effusion among patients with serum C-reactive protein ≥ 10 mg/dL.

Variable	Univariable Analysis		Multivariable Analysis ^a^	
	OR [95% CI]	*p*-Value	OR [95% CI]	*p*-Value
Blood tests				
WBC (cells/µL)	0.835 [0.752–0.927]	<0.001	0.951 [0.827–1.092]	0.474
CRP (mg/dL)	0.824 [0.749–0.908]	<0.001	0.983 [0.883–1.094]	0.754
Pleural fluid analysis				
pH	150.8 [7.7–2941.9]	<0.001	145.4 [1.6–12,926.8]	0.050
WBC (cells/µL)	1.000 [1.000–1.000]	0.026	1.000 [1.000–1.000]	0.108
PMN (%)	0.931 [0.903–0.960]	<0.001	0.989 [0.948–1.031]	0.593
ADA (IU/L)	1.017 [1.007–1.027]	0.001	1.053 [1.029–1.079]	<0.001
Radiological findings				
Loculated	0.127 [0.046–0.347]	<0.001	0.307 [0.062–1.520]	0.148
Pulmonary nodular lesions	29.570 [10.637–82.197]	<0.001	21.853 [5.210–91.652]	<0.001

OR = odds ratio; CI = confidence interval; WBC = white blood cell; CRP = C-reactive protein; PMN = polymorphonuclear leukocyte; ADA = adenosine deaminase. ^a^ After excluding cases with missing data, the analytic sample size was *n* = 343 (Group A, *n* = 20; Group C, *n* = 323) for the multivariable model.

**Table 3:** Diagnostic performance of independent predictors and composite model for discriminating polymorphonuclear leukocyte-predominant tuberculous pleural effusion from parapneumonic pleural effusion among patients with serum C-reactive protein ≥ 10 mg/dL.

Parameter	Cutoff	Sensitivity (%) [95% CI]	Specificity (%) [95% CI]	PPV (%) [95% CI]	NPV (%) [95% CI]	AUROC [95% CI]
Independent predictors (from multivariable analysis)						
PF ADA (IU/L)	>55 ^a^	70.0 [48.1–85.5]	78.5 [73.84–82.6]	16.3 [10.0–25.5]	97.8 [95.2–99.0]	0.798 [0.683–0.889]
Pulmonary nodular lesions	Present	70.0 [48.1–85.5]	93.0 [89.9–95.4]	37.8 [24.1–53.9]	98.1 [95.9–99.1]	0.817 [0.713–0.916]
Composite two-tier decision rule ^b^						
Pulmonary nodular lesions or PF ADA > 80 IU/L if nodular lesions absent	—	80.0 [58.4–91.9]	86.2 [82.1–89.5]	25.8 [16.6–37.9]	98.6 [96.5–99.5]	0.925 ^c,d^ [0.881–0.974]

CI = confidence interval; PPV = positive predictive value; NPV = negative predictive value; AUROC = area under the receiver operating characteristic curve; PF = pleural fluid; ADA = adenosine deaminase. ^a^ Optimal cutoff derived from the Youden index (sensitivity + specificity − 1) applied to the ROC curve for PF ADA alone. ^b^ Derived by algebraic simplification of the multivariable Firth logistic regression model combining PF ADA and pulmonary nodular lesions (composite score = −4.874 + 0.0172 × PF ADA + 3.351 × nodular lesion, transformed via the logistic function); the cutoff for the composite probability was selected using the Youden index. This decision boundary is mathematically equivalent to the two-tier rule shown. ^c^ Optimism-corrected AUROC (Harrell’s bootstrap method, 500 resamples); the apparent (non-corrected) AUROC was unchanged at 0.925, indicating minimal overfitting for this 2-predictor model. ^d^ The composite AUROC was significantly higher than PF ADA alone (difference 0.126, *p* = 0.016) and pulmonary nodular lesions alone (difference 0.109, *p* = 0.003) by the DeLong test for correlated ROC curves.

## Data Availability

The datasets used and/or analyzed during the present study are available from the corresponding author on reasonable request.
